# Carbapenemase Genes among Multidrug Resistant Gram Negative Clinical Isolates from a Tertiary Hospital in Mwanza, Tanzania

**DOI:** 10.1155/2014/303104

**Published:** 2014-02-24

**Authors:** Martha F. Mushi, Stephen E. Mshana, Can Imirzalioglu, Freddie Bwanga

**Affiliations:** ^1^Department of Microbiology, Catholic University of Health and Allied Sciences, P.O. Box 1464, Mwanza, Tanzania; ^2^Department of Medical Microbiology, School of Biomedical Sciences, Makerere University College of Health Sciences, P.O. Box 7072, Kampala, Uganda; ^3^Institute of Medical Microbiology, Justus-Liebig University, Schubertstraße 81, 35392 Giessen, Germany; ^4^German Centre for Infection Research (DZIF), Partner Site Giessen-Marburg-Langen, Campus, Giessen, Germany; ^5^MBN Clinical Laboratories, Plot 28 Nakasero Road, Kampala, Uganda

## Abstract

The burden of antimicrobial resistance (AMR) is rapidly growing across antibiotic classes, with increased detection of isolates resistant to carbapenems. Data on the prevalence of carbapenem resistance in developing countries is limited; therefore, in this study, we determined the prevalence of carbapenemase genes among multidrug resistant gram negative bacteria (MDR-GNB) isolated from clinical specimens in a tertiary hospital in Mwanza, Tanzania. A total of 227 MDR-GNB isolates were analyzed for carbapenem resistance genes. For each isolate, five different PCR assays were performed, allowing for the detection of the major carbapenemase genes, including those encoding the VIM-, IMP-, and NDM-type metallo-beta-lactamases, the class A KPC-type carbapenemases, and the class D OXA-48 enzyme. Of 227 isolates, 80 (35%) were positive for one or more carbapenemase gene. IMP-types were the most predominant gene followed by VIM, in 49 (21.59%) and 28 (12%) isolates, respectively. Carbapenemase genes were most detected in *K. pneumoniae* 24 (11%), followed by *P. aeruginosa* 23 (10%), and *E. coli* with 19 isolates (8%). We have demonstrated for the first time a high prevalence of MDR-GNB clinical isolates having carbapenem resistance genes in Tanzania. We recommend routine testing for carbapenem resistance among the MDR-GNB particularly in systemic infections.

## 1. Introduction

Carbapenem antibiotics have been used as the last resort salvage treatment for infections caused by multidrug resistance-gram negative bacteria (MDR-GNB) [1], that is, gram negative bacteria resistant to at least three of the following antimicrobials: ampicillin, augmentin, ceftazidime, ciprofloxacin, gentamicin, and/or trimethoprim-sulfamethoxazole (SXT) [2]. Thus, resistance to carbapenems becomes a real threat to the survival of patients with infections caused by MDR-GNB, and the overall mortality in such infections has been reported to be up to 50% [3, 4].

Resistance to carbapenems among the MDR-GNB is mostly due to the production of carbapenemases, which are *β*-lactamases with capacity to hydrolyze not only the carbapenems themselves but also all the other beta lactam agents [5]. Some of these carbapenemases include veron integron metallo-beta-lactamases, imipenemase, *Klebsiella pneumoniae* carbapenemases, oxacillinase-48, and New Delhi metallo-beta-lactamase_1 which are encoded by what is termed carbapenem resistance determining genes (CRDG): *bla*_VIM_, *bla*_IMP_, *bla*_KPC_, *bla*_OXA-48_, and *bla*_NDM_, respectively [6].

Recently, increasing resistance to carbapenems in health care associated infections has been reported worldwide [1, 7]. Studies in New York City found 39% of patients with fecal colonization of *KPC* producing *K. pneumoniae* [3]. In Africa, data on the prevalence and distribution of carbapenem resistance among the MDR-GNB is still limited. Few studies have been found to report on this problem; a surveillance study done in Kenya reported the recovery of seven NDM-1-positive *Klebsiella pneumoniae* isolates mostly from urine samples [8]. While in two other studies the prevalence of metallo-beta-lactamase among *Pseudomonas aeruginosa* was reported to be 14% in Kenya [9] and 67% in Northern Africa [10]. Other studies in South Africa reported the existence of carbapenemase producers among ESBL isolates [4, 11].

In this study, we determined a wide range of carbapenem resistance determining genes (*bla*_VIM_, *bla*_IMP_, *bla*_KPC_, *bla*_OXA-48_, and *bla*_NDM_) among different MDR-GNB isolates from patient specimens in Bugando Medical Centre—a tertiary hospital in Mwanza, Tanzania. All bacterial species which were resistant to three or more classes of antibiotics were regarded as MDR-GNB and included in the study.

## 2. Materials and Methods

### 2.1. Study Design and Population

This was a cross-sectional laboratory based study involving 234 multidrug resistant gram negative isolates collected between 2007 and 2012 from clinical specimens in a tertiary hospital, Northwestern, Tanzania. These isolates were from pus (112), urine (56), blood (55), aspirate (3), and sputum (1). The primary culture, identification using biochemical tests, and disk diffusion susceptibility testing of these isolates were done at Bugando Medical Centre following previously published techniques [12, 13]. All the isolates had been confirmed to be resistant to ampicillin, and 177 (78%) were ESBL producers as confirmed by double disk synergy test [13] and were frozen in brain heart infusion (BHI) broth with 20% glycerol at minus 80°C at the Microbiology Laboratory of Bugando Medical Centre.

### 2.2. Subculturing and Disk Diffusion Susceptibility Testing

Isolates were subcultured on blood agar (BA) and then resubjected to further susceptibility testing on Muller-Hinton agar to ampicillin 25 *μ*g, amoxicillin/clavulanic acid 20/10 *μ*g, ceftazidime 30 *μ*g, ciprofloxacin 5 *μ*g, gentamicin 10 *μ*g, trimethoprim-sulfamethoxazole (TMP/SMX) 1.25/23.75 *μ*g, ertapenem 10 *μ*g, and meropenem 10 *μ*g (Oxoid, UK). All susceptibility results were interpreted based on the CLSI 2010 guidelines [14].

### 2.3. PCR Amplification for Carbapenemase Genes

All the molecular/PCR tests (DNA extraction, amplification, and gel electrophoresis were conducted at MBN Clinical Laboratories, Kampala, Uganda. The presence of carbapenemase encoding genes was determined using primers targeting *bla*_VIM_, *bla*_IMP_, *bla*_KPC_, *bla*_OXA-48_, and *bla*_NDM_ [15], obtained from Eurofin MWG Operon, Germany, as shown in Table 1. Cells were lysed using boiling method to obtain both genomic and plasmid DNA as described previously [16]. For amplification, 5 *μ*L of template DNA (50 ng/*μ*L) was added to a 45 *μ*L mixture containing 200 *μ*M of dNTP mixtures (Roche, Switzerland), 0.4 *μ*M of each primer, 2.5 U Taq polymerase (Invitrogen, Germany), and appropriate buffer (0.2 *μ*M MgCl_2_, 2.5 *μ*M KCL, 0.5 *μ*L 10% Tween 20, 1 *μ*L of Gelatin, and 3.8 *μ*L of pure water).

The amplification was done using GTQ-CYCLER 96 thermocycler machine (Hain Life science GmbH, Nehren, Germany). For *bla*_VIM_, *bla*_KPC_, *bla*_NDM_, and *bla*_OXA-48_, the programme was denaturation at 94°C for 45 seconds, annealing at 52°C for 1 minute, and elongation at 72°C for a minute. For *bla*_IMP_ the same programme was used except that the annealing temperature was adjusted to 45°C for 60 seconds. The cycles were repeated 40 times and all primer sets had a final extension of 72°C for 10 minutes.

Five micro liters of PCR products were analyzed by electrophoresis in 1.0% agarose stained with ethidium bromide to detect the specific amplified product by comparing with 100 base-pairs standard DNA ladder (Promega, German). Quality control was performed with each run using DSMZ 9377 *Klebsiella pneumoniae* as the negative control for all genes. Positive control strains from the Institute of Microbiology, Giessen, Germany, were *Klebsiella pneumonia* Nr.8 for NDM-1, *Klebsiella pneumoniae* 714 for OXA-48, *Klebsiella pneumoniae* 211 (T) for KPC, and *P. aeruginosa* from clinical routine samples for IMP in Giessen. For the VIM gene, the control strain was obtained from RESET research collaboration [17].

### 2.4. Ethical Issues

The study was approved by the school of biomedical sciences research and ethics committee of Makerere University College of Health Sciences. Material transfer agreement for transportation of 234 isolates from Mwanza, Tanzania, to Kampala, Uganda, was obtained from the director of research and publication Catholic University of Health and Allied Sciences, Bugando.

## 3. Results

### 3.1. Clinical Bacterial Isolates Studied

The study excluded 9 isolates which failed to grow on subculture. Of the remaining 227 isolates which successfully grew, 76 (33.5%) were *K. pneumoniae* as the most predominant species followed by *E. coli* 56 (24.7%) and *P. aeruginosa* 41 (18.1%) (Table 2). The study used isolates which were resistant to at least three different classes of antibiotics; most of them were resistance to ampicillin 100%, augmentin, and ceftazidime (Table 3). For this study, strains displaying breakpoints for either resistance or intermediate levels for ertapenem and meropenem were considered as reduced susceptible. Of the 227 MDR-GNB, 55 (24%) had reduced susceptibility to ertapenem while only 15 (7%) had reduced susceptibility to meropenem.

### 3.2. Prevalence of Carbapenemase Genes

Based on the PCR assays, 80 (35.24%) of 227 MDR-GNB isolates were positive for one or more of the carbapenemase genes. Of the 55 isolates with reduced susceptibility to ertapenem, 33 (60%) tested positive for carbapenemase genes (*P* value < 0.001) while 11 (73%) of the 15 isolates with reduced susceptibility to meropenem were positive for carbapenemase genes (*P* value = 0.001).

Overall, IMP-types were the most predominant carbapenemase genes detected in 49 (21.6%), followed by VIM 28 (12.3%), OXA-48 11 (4.9%), KPC 8 (3.5%), and NDM 7 (3.1%) (Table 4). These genes were either solitarily detected in one bacterial isolate or with more than one gene in one bacterial isolate. Of 80 bacterial isolates with carbapenemase genes, 15 (6.6%) harbored more than one carbapenemase gene (Table 5).

### 3.3. Distribution of Carbapenemase Genes among Multidrug Resistant Gram Negative Bacterial Isolates

The genes were heterogeneously distributed among the different species of multidrug resistant gram negative bacteria with some bacteria species having more than one carbapenemase genes as shown in Table 5. *E. coli *was the most prevalent species with carbapenemase genes 32 (14%), followed by *Klebsiella pneumoniae* 24 (10.57%), *P. aeruginosa* 10.13%, *Klebsiella oxytoca* 1.76%, *Acinetobacter baumannii* 1.3%, *Citrobacter freundii* 0.88%, *Serratia marcescens *0.88%, and Salmonella spp. 0.44% (Table 4). Of the clinical specimens studied, carbapenemase genes were more prevalent in urine cultures 22 (39.29%) of 56 specimens followed by blood culture 20 (36.36%) of 55 specimen and pus swab with 37 (33.04%) of 112 specimen studied.

## 4. Discussion

We detected a high prevalence (35.24%) of carbapenemase genes among multidrug resistant gram negative bacterial species. The majority of the studied isolates were ESBL producer; thus, our results are similar to those obtained by Coetzee and Brink in South Africa [11]. This is a rather worrying finding in the poor populations in the horn of Africa; however, this data is comparable to study done in India which found a similar prevalence, particularly of MBL (NDM_1, VIM types, and IMP types) among family Enterobacteriaceae. In their study the prevalence of MBL was between 31% and 55% among multidrug resistant family Enterobacteriaceae [18]. This prevalence is in concordance with another study obtained in India which reported the prevalence of carbapenemase genes among gram negative bacterial isolates to be 43% [19, 20]. On the other hand, this magnitude is a bit higher than data reported from USA and Kenya [9, 21]; this could be explained by the fact that those studies investigated fewer genes than in the current study.

In the current study, we also detected 22 bacterial species with phenotypically reduced susceptibility to carbapenem drugs but its resistance mechanisms were not detected by any of the screened carbapenemase primers used in this study. This might be due to the limited number of genes targeted in our study as well as to other mechanisms of resistance such as porin loss/mutations [22, 23].

As previously published, OXA-48 gene for carbapenem resistance has been found in ESBL producers especially those harboring CTX-M [24]. This was also proved in this study as most of OXA-48 gene was detected on CTX-M producing *K. pneumoniae* and *E. coli *[25, 26].

In comparison with other carbapenem-resistant genes *bla*_VIM_ poses the broadest range of substrate hydrolysis and can eventually degrade all *β*-lactam except monobactams [27]. In the present study, these genes were mostly detected in* K. pneumoniae, E. coli,* and *P. aeruginosa. *This data corresponds to the findings of a study done in Korea where VIM was reported as the most predominant carbapenemase genes in class B metallo-beta-lactamase among gram negative clinical isolates. It also corresponds to the worldwide findings where VIM is reported as the commonest MBL to be found [28].

We have also detected a low prevalence (*n* = 7/3.08%) of NDM gene among multidrug-resistant gram negative bacteria. This prevalence is much lower than the one reported in India by Kumarasamy et al. among convenience sample of family Enterobacteriaceaein which they obtained a prevalence of 31% to 55% [18]. Plasmids carrying carbapenemase genes like NDM-1 are diverse and can harbor a high number of additional resistance genes (e.g., ESBL-alleles) as well as other carbapenemase genes like Oxacillinase-48 types, VIM types, and so forth, as the source of multidrug resistance in one single bacteria [18, 29]. Of 80 bacterial species detected of having carbapenemase genes, 15 had multiple genes coding for carbapenem resistance especially in *E. coli* and *P. aeruginosa*. The presence of multiple resistance genes in one strain provides selection advantage of these strains [26]. This phenomenon has not been commonly detected in a large number of studies probably due to the number of genes studied since most of the studies research on one or two genes.

The study did not investigate the clonality of the isolates and the sequence of the genes and also did not use primers to target all known carbapenemases genes. Thus, there is a probability that some carbapenemase-producing isolates could not be adequately characterized. Despite these limitations, the study has provided the distribution of the common carbapenemase genes and the magnitude of the problem.

## 5. Conclusion and Recommendation

We have, for the first time, demonstrated a high prevalence of carbapenem-resistance conferring genes among multidrug resistant gram negative bacteria in Tanzania. Most of the isolates harboring carbapenemase genes originated from blood culture specimens and pus. We recommend routine testing for carbapenem resistance among the MDR-GNB in our hospital and other health facilities in developing countries where there is high prevalent MDR GNB. In addition, other antibiotics such as colistin and tigecycline should be tested to provide alterative treatment to these isolates. More studies should be done to determine evolution and molecular epidemiology of these isolates.

## Figures and Tables

**Table 1:** Primer sets for amplification of carbapenem resistance determine genes (16).

Gene	Primer sequence (5′→ 3′)	TM (°C)	Amplicons size (bp)
*bla*-_VIM_	Forward: GATGGTGTTTGGTCGCATA Reverse: CGAATGCGCAGCACCAG	54.5 57.6	390
*bla*-_KPC_	Forward: CATTCAAGGGCTTTCTTGCTGC Reverse: ACGACGGCATAGTCATTTGC	60.3 57.3	498
*bla*-_NDM_	Forward: GGTTTGGCGATCTGGTTTTC Reverse: CGGAATGGCTCATCACGATC	57.3 59.4	521
*bla*-_IMP-1_	Forward: TTGACACTCCATTTACAG Reverse: GATTGAGAATTAAGCCACTCT	49.1 54.0	232
*bla*-_IMP-2_	Forward: TTGACACTCCATTTACGG Reverse: GATCGAGAATTAAGCCACCCT	51.4 57.9	232
*bla*-_IMP-3_	TTGACACTCCATTTACTG GATCGAGAATTAAGCCACTCT	49.1 55.9	232
*bla*-_OXA-48_	Forward: GCTTGATCGCCCTCGATT Reverse: GATTTGCTCCGTGGCCGAAA	56.0 59.4	238

TM: melting temperature of the primer.

**Table 2:** Clinical isolate by specimens studied.

Bacteria spp.	Specimen					Total
	Aspirate	Blood	Pus swab	Sputum	Urine	
*K. pneumoniae *	1	23	28	0	24	76
*E. coli *	1	8	31	1	15	56
*P. aeruginosa *	1	16	22	0	2	41
*C. freundii *	0	1	8	0	6	15
* A. baumannii *	0	2	8	0	0	10
*P. vulgaris *	0	0	7	0	0	7
*E. cloacae *	0	3	0	0	2	5
*M. morganii *	0	0	2	0	1	3
*P. mirabilis *	0	0	1	0	1	2
*S. marcescens *	0	2	4	0	3	9
*Salmonella typhi*	0	0	0	0	1	1
*Salmonella* spp.	0	0	1	0	1	2
Total	**3**	**55**	**112**	**1**	**56**	**227**

**Table 3:** Resistance pattern of bacteria species used in the study.

Isolate	AMP	AMC	CRO	CAZ	CN	CP	SXT	ERT	MEM
*A. baumannii * (10)	100.0%	100.0%	90.0%	90.0%	80.0%	40.0%	90.0%	40.0%	10.0%
*C. freundii *(15)	100.0%	100.0%	60.0%	80.0%	80.0%	26.7%	86.7%	13.3%	0.0%
*E. coli* (56)	100.0%	100.0%	84.0%	82.1%	73.2%	44.6%	96.4%	19.6%	8.9%
*Enterobacter *(5)	100.0%	100.0%	20.0%	40.0%	60.0%	20.0%	100.0%	20.0%	0.0%
*K. oxytoca * (8)	100.0%	100.0%	100.0%	100.0%	100.0%	62.5%	100.0%	12.5%	0.0%
*K. pneumoniae *(68)	100.0%	98.5%	84.0%	79.4%	83.8%	33.8%	97.1%	16.2%	1.5%
*M. morganii *(3)	100.0%	100.0%	33.3%	33.3%	33.3%	0.0%	100.0%	0.0%	0.0%
*P. aeruginosa *(41)	100.0%	97.5%	73.2%	53.7%	31.7%	17.1%	92.7%	56.1%	19.5%
*P. mirabilis *(2)	100.0%	100.0%	100.0%	100.0%	50.0%	100.0%	100.0%	50.0%	0.0%
*P. vulgaris *(7)	100.0%	85.7%	57.1%	42.9%	57.1%	14.3%	85.7%	14.3%	0.0%
*S. marcescens *(9)	100.0%	100.0%	66.7%	66.7%	66.7%	44.4%	100.0%	0.0%	0.0%
*Salmonella* spp. (3)	100.0%	100.0%	100.0%	100.0%	100.0%	0.0%	100.0%	0.0%	0.0%
Total	**100.0%**	**98.7%**	**78.0%**	**74.0%**	**65.6%**	**33.5%**	**95.2%**	**24.2%**	**6.6%**

AMP, AMC, CAZ, CRO, CN, CIP, SXT, ERT, and MEM stand for ampicillin, amoxicillin/clavulanic acid, ceftazidime, gentamicin, ciprofloxacin, trimethoprim-sulfamethoxazole, ertapenem, and meropenem, respectively.

**Table 4:** Distribution of carbapenemase genes among different organisms studied.

Bacterial spp.	Carbapenemase genes					
	IMP_types	VIM	OXA_48	KPC	NDM	Carbapenemase positive
*A. baumannii *(10)	3	0	0	0	0	3
*C. freundii *(15)	2	1	1	0	0	4
*E. coli *(56)	19	4	3	4	2	32
*E. cloacae *(5)	0	0	0	0	0	0
*K. oxytoca * (8)	3	0	1	0	1	5
*K. pneumoniae *(68)	9	11	4	3	2	29
*M. morganii *(3)	0	0	0	0	1	1
*P. aeruginosa *(41)	12	9	2	1	1	25
*P. mirabilis *(2)	0	0	0	0	0	0
*P. vulgaris *(7)	0	0	0	0	0	0
*S. marcescens *(9)	0	2	0	0	0	2
*Salmonella* spp. (3)	1	1	0	0	0	2
	**49**	**28**	**11**	**8**	**7**	**103**

**Table 5:** Antimicrobial susceptibility profile of isolates with multiple carbapenem resistance genes.

	Isolate	CAZ	CRO	AMC	AMP	CN	CIP	ERT	MEM	SXT	Carbapenemase genes
1	*K. pneumoniae *	R	R	R	R	R	S	R	S	R	KPC, IMP, VIM
2	*E. coli *	R	R	R	R	S	R	R	R	R	KPC, IMP
3	*K. pneumoniae *	R	R	R	R	R	S	S	S	R	OXA_48 and VIM
4	*P. aeruginosa *	R	R	R	R	S	S	R	S	R	IMP_C and VIM
5	*E. coli *	R	R	R	R	R	R	S	S	R	KPC and IMP
6	*K. pneumoniae *	R	R	R	R	R	R	S	S	R	IMP and VIM
7	*K. pneumoniae *	R	R	R	R	R	S	S	S	R	IMP and VIM
8	*E. coli *	R	R	R	R	R	R	S	S	R	IMP and VIM
9	*E. coli *	R	R	R	R	R	R	S	S	R	OXA_48, IMP
10	*E. coli *	R	R	R	R	R	S	R	S	R	OXA_48 and NDM
11	*E. coli *	R	R	R	R	S	S	S	S	R	VIM and NDM
12	*K. pneumoniae *	R	R	R	R	R	R	R	S	R	OXA_48 and VIM
13	*E. coli *	R	R	R	R	R	R	R	R	R	IMP and VIM
14	*C. freundii *	R	R	R	R	R	S	S	S	R	OXA_48, IMP and VIM
15	*K. oxytoca *	R	R	R	R	R	S	R	S	R	OXA_48 and NDM
